# Experimental evolution of viruses: Microviridae as a model system

**DOI:** 10.1098/rstb.2010.0053

**Published:** 2010-08-27

**Authors:** Holly A. Wichman, Celeste J. Brown

**Affiliations:** Department of Biological Sciences, University of Idaho, Moscow, ID, USA

**Keywords:** experimental evolution, adaptive evolution, bacteriophage, virus

## Abstract

φX174 was developed as a model system for experimental studies of evolution because of its small genome size and ease of cultivation. It has been used extensively to address statistical questions about the dynamics of adaptive evolution. Molecular changes seen during experimental evolution of φX174 under a variety of conditions were compiled from 10 experiments comprising 58 lineages, where whole genomes were sequenced. A total of 667 substitutions was seen. Parallel evolution was rampant, with over 50 per cent of substitutions occurring at sites with three or more events. Comparisons of experimentally evolved sites to variation seen among wild phage suggest that at least some of the adaptive mechanisms seen in the laboratory are relevant to adaptation in nature. Elucidation of these mechanisms is aided by the availability of capsid and pro-capsid structures for φX174 and builds on years of genetic studies of the phage life history.

## 1. Introduction

Humans have long dabbled with selection—both intentionally and unintentionally. Indeed, the process of artificial selection was used by Darwin to illustrate the process of natural selection (Darwin 1859). In the laboratory, experimental evolution has been used to investigate the patterns and mechanisms of evolution, and to test some underlying assumptions of population genetic theory. On a grander scale, our many interventions pose strong selective pressures in the natural world, and thus it is particularly useful to have model systems to study strong selection in real time. Such studies have been carried out on a wide array of organisms including, but not limited to, mice, fish, insects, bacteria and viruses. Yet, it is only recently that we have been able to deduce the underlying genetic basis of such laboratory adaptation, and we are only now beginning to investigate the mechanisms underlying adaptive change in these systems. Here, we consider what we have learned from one such model system, the Microviridae, with special emphasis on the prototype isolate φX174.

The Microviridae are tailless icosahedral bacteriophages with a single-stranded, circular DNA genome (Hayashi *et al*. 1988; Fane *et al*. 2006). Among the group that infects coliform bacteria, genome size ranges from 5.4 to 6.3 kb (Rokyta *et al*. 2006). In general, the genome encodes 11 genes, nine of which are essential (table 1), and has overlapping reading frames for several genes. Exceptions are microvirid phages isolated from the γ-proteobacteria, which are distantly related to the coliform microvirids and have smaller genomes, and members of the *α*3- and WA13-like phages, which have five additional conserved reading frames of unknown function (Rokyta *et al*. 2006). These differences will not be considered further here.

**Table 1.** Genes encoded by bacteriophages in the Microviridae^a,b^.

gene	protein encoded	essential?	function (number)
A	replication initiation	yes	stage II and stage III DNA replication
A*	shortened version of protein A	no	thought to inhibit host DNA replication; may be involved in superinfection exclusion
B	internal scaffold	yes^c^	procapsid morphogenesis (60 in procapsid)
C	DNA packaging	yes	switch from stage II to stage III; stage III DNA replication
D	external scaffold	yes	procapsid morphogenesis (240 in procapsid)
E	lysis	yes	facilitates host cell lysis
F	major capsid	yes	morphogenesis; host recognition (60 copies in procapsid and virion)
G	major spike	yes	morphogenesis; host recognition (60 copies in procapsid and virion)
H	minor spike (pilot)	yes	morphogenesis; host recognition; needed for DNA injection (12 copies in procapsid and virion)
J	DNA binding	yes	required for DNA packaging (60 copies in virion)
K		no	unessential; may affect burst size

^a^Taken from (Baas 1985; Hayashi *et al*. 1988; Fane *et al*. 2006).

^b^Five putative genes of unknown function found only in the *α*3-like and WA13-like phages (Rokyta *et al*. 2006) are not included.

^c^φX174 can be evolved to be independent of the internal scaffolding protein (Chen *et al*. 2007).

φX174 was developed as a model system for experimental studies of evolution by J. J. Bull during a sabbatical in the laboratory of Bruce Levin at Emory University in 1993–1994. This system was chosen for its small DNA genome, which facilitated whole genome sequencing, and for its ease of laboratory cultivation, which facilitated passaging large populations in a short period of time at relatively low cost. The first studies looked at the extent of parallel evolution in this system (Bull *et al*. 1997; Wichman *et al*. 1999), and subsequent studies have examined host-specific adaptation (Crill *et al*. 2000; Wichman *et al*. 2000; Pepin *et al*. 2008), temperature adaptation (Bull *et al*. 2000; Holder & Bull 2001; Knies *et al*. 2006, 2009), the number, size and distribution of beneficial mutations (Bull *et al*. 2000; Rokyta *et al*. 2005, 2008), pleiotropy (Pepin *et al*. 2006), epistasis (Bull *et al*. 2000; Pepin & Wichman 2007), evolutionary dynamics (Wichman *et al*. 1999, 2005; Holder & Bull 2001; Bull *et al*. 2006; Pepin & Wichman 2008; Dickins & Nekrutenko 2009), evolution of resistance (Cherwa & Fane 2009), compensatory evolution (Poon & Chao 2005, 2006), recombination (Wichman *et al*. 2005; Rokyta *et al*. 2006, 2009), domestication (Rokyta *et al*. 2009) and spatial dynamics (Coberly *et al*. 2009). Some of the later studies took advantage of a collection of wild isolates of the Microviridae (Rokyta *et al*. 2006), and increasingly they have taken advantage of the known structure of most proteins in the viral capsid (McKenna *et al*. 1992, 1994; Dokland *et al*. 1997).

## 2. Material and Methods

Molecular changes seen when φX174 was evolved in the laboratory were compiled from 10 experiments and a total of 58 lineages (Bull *et al*. 1997, 2006; Wichman *et al*. 1999, 2000, 2005; Crill *et al*. 2000; Pepin *et al*. 2008; Pepin & Wichman 2008; and M. W. Rain & H. A. Wichman 2001, unpublished data; J. Millstein & H. A. Wichman 2003, unpublished data). These experiments were carried out under a variety of conditions. Two studies comprising 16 lineages were carried out by flask passaging (Pepin & Wichman 2008; Pepin *et al*. 2008) while the remainder were carried out in chemostats. For flask evolution experiments, each passage was 30 min and total passage time ranged from 40 to 87 h; viral populations were smaller than host populations so that co-infection and recombination were minimized. Chemostat passages were carried out for 10–11 (34 lineages), 22 (3 lineages), 50 (4 lineages) or 180 days (1 lineage); viral populations were much larger than host populations and were characterized by frequent selective sweeps, and co-infection and recombination were common. Of the 58 lineages evolved, 18 used *Escherichia coli* C as the host, 16 used defined lipopolysaccharide (LPS) variants of *E. coli*, three used *Shigella* and 21 used *Salmonella*; two lineages were evolved at 32°C, 30 lineages at 37°C and 26 lineages at high temperature (42–43.5°C); 16 lineages were founded from our laboratory ancestor (GenBank accession AF176034), 15 from an isolate of this ancestor pre-adapted to flask passaging and 27 from previously-evolved chemostat lineages.

In the early studies using this system, a single isolate was sequenced at the end of an experiment (Bull *et al*. 1997; Wichman *et al*. 1999, 2000; Crill *et al*. 2000). However, in more recent studies multiple genomes were sequenced, and in many cases genomes were sequenced from multiple time points over the course of the study. This allowed for rough estimates of the frequency of a particular change in a population as well as observation of the dynamics of substitutions sweeping through the population. For any given lineage, a mutation was counted only once. For published studies, sequence data are available in GenBank or in tabular form in the publication. Data for one unpublished study can be found under GenBank accession numbers AF299300 through AF299314.

When summing over all 58 evolved lineages, a total of 667 changes were seen at 337 sites. A total of 508 amino acid substitutions were seen. Note that, because of overlapping reading frames, a single base substitution can affect more than one codon. To determine the probability of seeing substitutions at the same site in the absence of adaptive evolution, we conservatively assumed that 25 per cent of sites are invariant (i.e. subject to strong purifying selection). We thus used the following equation to calculate the number of sites at which we expect to see substitutions occurring *n* times
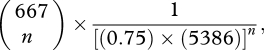

where 667 is the total number of substitutions seen in all experiments, (0.75) × (5386) is the estimated number of potentially variable sites in φX174 and *n* is the number of times a substitution occurs at the same site.

## 3. Results and discussion

### (a) Parallel and convergent evolution

Here, we define parallel evolution as independent evolution of the same molecular substitution from a common ancestor. We use the term convergent evolution to describe evolution of the same molecular substitution in two independent ancestors. In experimental evolution, where the ancestor is known and replicate adaptations are carried out, parallel evolution is easy to document. In nature it is not always trivial to distinguish between parallel and convergent evolution because the ancestral state is not usually known.

**Figure 1.**
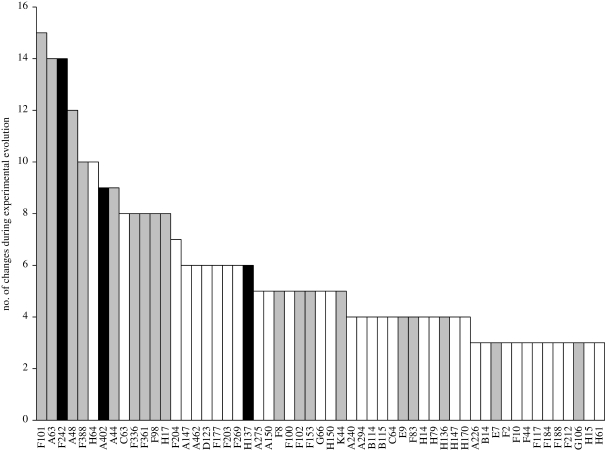
Common sites of an amino acid substitution during experimental evolution. Histogram shows the number of times an amino acid substitution was seen at each residue among the 58 experimental lineages analysed. Only amino acid residues with three or more substitutions are included. Grey bars indicate sites that also vary among wild φX-like phages (Rokyta *et al*. 2006 and unpublished isolates). Black bars indicate substitutions that converge on the sequence at residues that are invariant among the wild φX-like phages. In most cases, the substitutions were parallel events of the same substitutions from a common ancestor, but reversions and alternate substitutions at the same residue were also counted. However, some lineages were extensions of other experiments and thus already carried amino acid substitutions relative to the ancestor, so, depending on the selective environment, not all substitutions have an equal opportunity of arising.

One of the most pronounced characteristics of experimental evolution in this system is the high incidence of parallel evolution. For any two experiments carried out under the same experimental conditions, around 50 per cent of substitutions arose in both experiments, but there is still considerable parallel evolution between experiments carried out under different experimental conditions (Bull *et al*. 1997; Wichman *et al*. 2000; Pepin & Wichman 2008). Although evolution in the same gene is frequently seen during experimental evolution of organisms with larger genomes (Barrick *et al*. 2009; Harcombe *et al*. 2009), parallel evolution at the level of identical nucleotides is rare or uncommon. Even in the smaller genome of the RNA bacteriophage MS2, parallel evolution at the level of the nucleotide does not occur at the rate observed in this system (Betancourt 2009). Parallel evolution is often considered as evidence for adaptive evolution, but in this large dataset some parallel evolution is expected by chance alone. Given the number of substitutions observed and the genome size, two or more occurrences of substitutions at the same site are expected to occur by chance 55 times, accounting for 16 per cent of substitutions, but three or more occurrences at the same site are expected only three times (1% of substitutions). In the total dataset, over 50 per cent of substitutions occurred at sites with three or more events, and 67 per cent occurred at sites with two or more events. To be conservative, we consider only three or more events at the same site as strong evidence for adaptive substitution, and our subsequent discussion will focus mainly on these sites (figure 1). If we define parallel evolution in this case to be the same base substitutions at the same sites in the virus three or more times, parallel events occurred for 297 of the 667 substitutions. Thus, parallel evolution was pervasive, especially given that we are combining experiments carried out under varied conditions. However, it is important to note that where the dynamics of substitutions have been tracked in these experiments, it appears that many or most substitutions are adaptive (Wichman *et al*. 1999, 2005).

One might ask to what extent we can extrapolate the results of experimental evolution of viruses to adaptation of similar organisms in nature and to evolutionary processes in more complex genomes. One way to address this question is to compare the sites of variation among natural isolates to sites of adaptation during experimental evolution. If experimental evolution is using the same pathways of adaptation that are used in nature, we might expect to see an excess of sites in common between experimental and wild variants (i.e. evidence of parallel and convergent evolution).

Considering that amino acid substitutions were seen at 216 sites during experimental evolution (11% of the 1986 amino acid positions) compared with 206 variable amino acid positions among the wild phage (10%), we would expect 22 (1.1%) of the sites to be in common; we observed 47 in common. We can now consider only sites where we have strong evidence that substitutions in our experiments are adaptive. If we consider only the 58 amino acid sites where we saw evolution three or more times, there is variation at 36 per cent of these sites among the wild phage (figure 1). Evolution at these positions accounts for over 64 per cent of the 508 amino acid substitutions seen in our experiments. The pattern becomes even stronger if we consider only the most frequent substitutions. Some sites had a very high rate of substitution during experimental evolution. There were 13 amino acid positions at which changes occurred eight or more times among the experimental lineages, accounting for 26 per cent of the 508 amino acid substitutions seen. Variation or convergent evolution was seen among the wild phage at 11 of these 13 residues. Thus, there is strong evidence that at least some of the adaptive mechanisms seen in the laboratory are relevant to adaptation in nature.

There was also considerable variation at regulatory positions in the virus. There are 133 nucleotide positions that have been shown to regulate transcription or translation either experimentally or by sequence similarity to known regulatory motifs (Hayashi *et al*. 1988; Fane *et al*. 2006). Forty putatively adaptive substitutions and/or indels have arisen in response to selection at 17 of these positions (12%) in the sigma factor binding, transcription termination or ribosome binding sites.

### (b) Host recognition

φX174 attaches to the LPS of some rough strains of Gram-negative bacteria including some *E. coli*, *Salmonella typhimurium* and *Shigella sonnei*. Host attachment has a reversible stage followed by an irreversible stage, but it is not known if LPS is the host receptor for only one or for both of these stages. In early studies, host recognition sites for φX174 and S13 were mapped genetically to the pilot protein H (Sinsheimer 1968), the major spike protein G (Newbold & Sinsheimer 1970; Weisbeek *et al*. 1973) and the major capsid protein F (Tessman 1965; Dowell *et al*. 1981), but the identities of host-specific mutations within these proteins are unknown. Specific interactions of LPS from φX174-susceptible strains, but not resistant strains, have been shown for the proteins G and H (Inagaki *et al*. 2000; Kawaura *et al*. 2000). Thus, had we been forced to use a candidate gene approach to study host recognition in this system, we would have focused on the pilot protein H and the spike protein G where there was both genetic and biochemical evidence for a role in host recognition. We did observe a considerable amount of evolution in the pilot protein H. A total of 105 substitutions were observed at 48 amino acid sites in H. Gene H makes up 16 per cent of the protein-coding capacity of φX174, so the expected number of changes is 81, if changes are distributed evenly in the genome. However, we do not yet have direct evidence that any of these changes occurred in response to selection for host recognition. In contrast, we have observed little evolution in the major spike protein G. Amino acid substitutions occurred at only eight sites in G, and at only one site (G66) was there considerable parallel evolution. The same substitution occurred at G66 five times, but under different host conditions and different temperature regimes, so there is no indication that this substitution was specific to host recognition.

**Figure 2.**
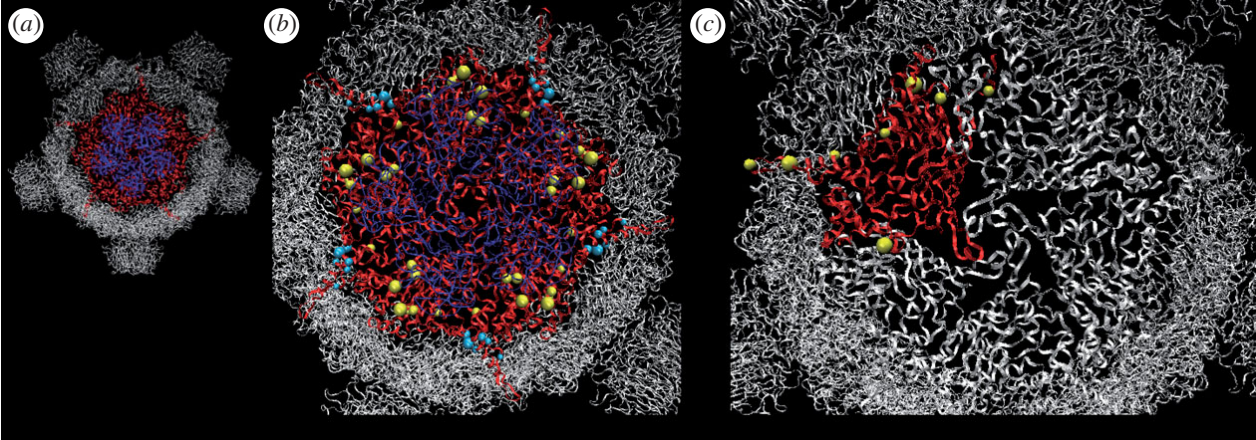
Structural location of select evolved sites. Images were rendered with VIPER using 1rb8_half.vdb from VIPERdb at http://viperdb.scripps.edu/. (*a*) Structure of the φX174 capsid. One of the 12 pentameric units is highlighted, with five copies of the major capsid protein F shown in red and five copies of the major spike protein G shown in blue. (*b*) Changes at sites of host recognition. Sites of host recognition in the major capsid protein F as suggested by experimental evolution on *Salmonella* are shown in yellow (F101, F102, F153 F336, F361, F364 and F388). The region of host recognition suggested by McKenna *et al*. (1994) based on the crystal structure are shown in aqua (F176, F178, F181, F205, F209 and F213). Amino acid number is based on GenBank accession AF176034. (*c*) Changes at sites of interaction between subunits of the major capsid protein. One copy of the major capsid protein F is highlighted in red. Experimentally evolved changes at or adjacent to sites of F–F interaction are shown in yellow (F2, F82, F115, F184, F188, F205, F227, F361 and F424).

Another piece of evidence suggesting a specific region for host attachment was the presence of sugar bound in a depression in the major capsid protein F of the crystal used to solve the atomic structure of φX174 (McKenna *et al*. 1994). Based on this ability to bind sugar and the location of host receptor sites in other viruses, McKenna *et al.* speculated that this was the host attachment region in the coat protein F. Although this six residue pocket (figure 2*b*) has been reported to be highly conserved, we observed variation both within and among coliform microvirid lineages. The region is conserved among all but two of the wild φX-like phage examined, but a total of nine haplotypes have been observed among the five previously sequenced laboratory strains and the wild isolates reported by Rokyta *et al.* (2006). In all haplotypes, the pocket consists of two to four negatively charged amino acids, zero to one positively charged amino acid, and one to three neutral amino acids, but charge is only conserved at one (neutral) position among all haplotypes. During experimental evolution, we saw only a single substitution at any of these six sites, and that change conserved the negative charge at the site. Some evolution occurred at adjacent sites but none of these substitutions caused a change in charge, and we do not know whether they affected phage interaction with LPS. Thus far, there is no experimental evidence for the involvement of this carbohydrate-binding pocket in host recognition or attachment.

Another region of the major capsid protein F was identified by experimental evolution to be important for host recognition. This region forms a raised ridge just under the lip of the capsid spike (figure 2). Evidence for the involvement of this region in host recognition includes: (i) direct measurement of the effect of substitutions at F101 and F102 in attachment (Crill *et al*. 2000; Pepin *et al*. 2006; Pepin & Wichman 2007); (ii) evolution and then reversion of substitutions at F101, F153, F336, F364 and F388 upon host switching (Crill *et al*. 2000); (iii) convergent evolution at F153 and F361 when the *Salmonella* phage S13 and the *E. coli* phage φX174 were evolved on alternate hosts (Wichman *et al*. 2000); and (iv) multiple independent substitutions at F101, F102, F153, F336 and F388 on *Salmonella* and at F100 on *E. coli*.

### (c) Temperature adaptation

Adaptation frequently involved reversion of the temperature-sensitive (ts) mutation common to some laboratory strains of φX174 (F242). This residue is a phenylalanine, not only in all of the φX-like phages we have characterized, but also in all of the other sequenced coliform microvirid phages (Rokyta *et al*. 2006); it is a leucine in our laboratory isolate and is the probable ts mutant in the φX174 isolate sequenced by Sanger (Sanger *et al*. 1977). This residue is in the *β*-barrel of the coat protein F, and substitution of phenylalanine for leucine confers the ability to grow at high temperature (Bull *et al*. 2000). Temperature was one of the selective pressures commonly used in experimental evolution, and the high temperature used in our experiments (42–43.5°C) is higher than what the phage are likely to be exposed to in the environments from which they were recovered. Fitness of our laboratory isolate at high temperature is negative (i.e. phage cannot produce a visible plaque or reproduce fast enough to maintain a constant population size under standard liquid passaging), so we would expect strong selection for not only the change at F242, but for other changes that stabilize the capsid. For example, adaptation to high temperature can also occur by evolution of scaffolding proteins. A change in the internal scaffolding protein at B114 confers the ability to grow at high temperature (Bull *et al*. 2000). This same mutation was seen in four experimental lines, always at high temperature. The only high-frequency amino acid substitution in the external scaffolding protein is at D123, which is a site of intra-dimer contact in the scaffold. This phenylalanine to leucine substitution occurred five times, always at high temperature.

We see considerable evolution at or immediately adjacent to known sites of protein–protein interaction (McKenna *et al*. 1994) between subunits of the coat protein F within the capsid (F2, F82, F115, F184, F188, F205, F227, F361 and F424) and at other sites near these regions of protein–protein interaction. While it is tempting to interpret these as adaptations to high temperature, changes at these sites actually occurred more frequently at 37°C. High temperature is not the only selective force that might be expected to affect protein–protein interactions. Because many adaptive mutations destabilize protein structure, compensatory mutations are frequently stabilizing (DePristo *et al*. 2005). Thus, many of these mutations at sites of protein–protein interaction may be compensatory for other adaptive but destabilizing mutations. Another possibility is that these mutations act on the capsid assembly, perhaps by suppressing off-pathway assembly products.

## 4. Conclusions

We compared evolution in the laboratory to variation in the wild. Overall, there was a strong signature of purifying selection among the wild phage. Excluding regions with out-of-frame overlaps in genes, silent differences were 3.8 times more prevalent than missense differences. This might be taken to suggest that most or all of the amino acid variation among the wild phage is neutral, but the considerable convergence at sites of adaptive evolution among the experimental lines suggests that some of this variation is a signature of adaptation in the wild. For example, all six of the sites in the major coat protein F identified as putative host recognition sites by experimental evolution vary among wild isolates. In contrast, of the 14 sites at or adjacent to known sites of interactions between F subunits, variation was seen among wild phage at only 5 sites. While this is more than would be expected by chance, the amount of variation at these sites is less than at host recognition sites. This is especially noteworthy because three of these five sites were also identified as potential host-interacting sites, so any signature of adaptive evolution in the wild may well be driven by host adaptation.

Certainly, the small genome size and limited number of proteins encoded contribute to the magnitude of parallel evolution in this experimental system and convergence between laboratory evolved strains and wild isolates. However, it is becoming increasingly clear that parallel and convergent evolution are also common in nature, especially when the target of selection is small (Wood *et al*. 2005). In some cases, convergent evolution at the molecular level has been seen between quite divergent species. For example, there are several examples of the evolution of insecticide resistance in different species of insects owing to identical amino acid changes in the same gene (Ffrench-Constant 1994; Feyereisen 1995; Ffrench-Constant *et al*. 2000). On the other hand, adaptive loss or down regulation of function frequently involves different mutations in the same gene; this has been shown for pigmentation genes in both flowers and fish, for instance Gross *et al*. (2009) and Streisfeld & Rausher (2009). Thus, even in complex organisms, the target of selection may be small.
